# Maternal influences on oral microbiome development and implications for early childhood health: a systematic review

**DOI:** 10.1128/iai.00585-25

**Published:** 2026-03-03

**Authors:** Audrey Wang, Lien Quynh Dao, Francisco Ramos-Gomez, Yan Wang

**Affiliations:** 1 Department of Psychobiology, University of California Los Angeles 8783, Los Angeles, California, USA; 2 School of Public Health, UC Berkeley 40289, Berkeley, California, USA; 3 Section of Pediatric Dentistry, School of Dentistry, University of California Los Angeles 8783, Los Angeles, California, USA; 4 Section of Public and Population Health, School of Dentistry, University of California Los Angeles 8783, Los Angeles, California, USA; University of California San Diego School of Medicine, La Jolla, California, USA

**Keywords:** oral microbiome, mother, infant, microbiome development, longitudinal

## Abstract

A deeper understanding of how maternal-infant interactions shape the establishment and diversification of the oral microbiome could have significant clinical applications; however, relatively few studies emphasize early maternal-infant microbial connections. This systematic review provides a longitudinal analysis of oral microbiome development from birth to five years, focusing on the relationship between maternal and infant microbiomes. We conducted a systematic search (June 2025) using keywords “mother,” “children,” “oral microbiome,” and “longitudinal” across PubMed, Cochrane Library, and Embase. Twelve studies fulfilled the inclusion criteria: longitudinal design, healthy mother-child dyads, saliva sample collection, and relevant age range. We excluded review articles, non-English publications, and studies with overlapping data. Results were synthesized by developmental stage and topic. Overall, current literature agrees that the mother is an important source of exposure for initial colonization of the newborn’s oral microbiome. Several studies indicated that the oral microbiome at birth is diverse and unspecialized, composed mostly of maternally derived strains. Rapid selection occurs over the first few weeks, as the relative abundances of typical oral bacterial species increase. Throughout the first year, increases in diversity strengthen the resemblance between infant and maternal microbiomes. The microbiome appears to stabilize around 3–5 years. In conclusion, maternal-infant connections play a significant role in influencing oral microbiome development during the first 5 years of life. This review highlights the need for future studies to incorporate larger, longitudinal designs with metadata and advanced tools to clarify the roles of delivery mode, tooth eruption, and parental lifestyle habits in shaping early oral microbiome development.

## INTRODUCTION

The oral microbiome comprises the complex community of microorganisms, including bacteria, fungi, viruses, archaea, and protozoa, that inhabit the human oral cavity (1). A healthy oral microbiome contains a balanced community of microorganisms that maintains ecological stability and prevents the proliferation of pathogenic species (2). As the initial site of digestion, the oral cavity acts as a first point of contact between the internal body systems and the external environment (1). Therefore, this microbial community plays a crucial role in both oral and systemic immune health (1, 3). Community imbalances have been linked to oral conditions such as dental caries, gingivitis, and periodontitis, as well as systemic diseases such as cardiovascular disease, diabetes, adverse pregnancy outcomes, and respiratory infections (1, 4, 5).

Due to its significant influence on immune health, the oral microbiome has emerged as a central focus in contemporary medical research. Several recent studies have demonstrated that dysbiosis, or imbalance, of the oral microbiome contributes to systemic inflammation, which may, in turn, lead to serious health complications (4–8). For example, oral dysbiosis has been linked to periodontal disease, a severe gum disease caused by the overgrowth of pathogenic bacteria. If left untreated, these periodontal pathogens can cause local tissue destruction and release harmful byproducts that spread beyond the oral cavity (5). These byproducts have been associated with numerous systemic health complications, including adverse pregnancy outcomes, cardiovascular disease, diabetes, respiratory disorders, and cancers (5). The establishment of a healthy oral microbiome early in life is particularly crucial, as the oral microbiome can also influence the child’s neurodevelopment and the progression of chronic diseases (5, 9). One study found an association between oral microbiome health and infants’ neurodevelopment, possibly affecting cognitive development (2). In addition, research done by Luppi et al. (4) suggests that oral pathogens may translocate to the gut, potentially contributing to the development of Type I Diabetes Mellitus in children (4). These findings collectively underscore the impact of the oral microbiome on children’s immunological health.

Exploring the maternal-infant relationship is key to understanding the early stages of oral microbiome development because the mother plays a vital role in the infant’s initial bacterial colonization. Although it is still debated whether the infant’s first exposure to bacteria begins before or after birth, it is well established that the mother is the infant oral microbiome’s primary source of colonizing bacteria (10). Some studies have reported oral microbes in the amniotic fluid of up to 70% of pregnant women, suggesting possible microbial transfer to the infant during birth through the uterus and vaginal canal (1, 11). After birth, breastfeeding and skin-to-skin contact further initiate microbial transfer (1, 11). As a result of these interactions, 6-month-old infants possess oral microbiomes remarkably similar to their mother’s, particularly the breast milk microbiota (12, 13).

The mother’s lifestyle and socioeconomic status (SES) can also impact the development of her child’s oral microbiome (14, 15). One way studies have measured this impact is through the occurrence of dental caries, a disease driven by acid-producing bacteria that causes tooth enamel and dentin to decay over time (14, 15). Dental caries are particularly relevant in infants and young children, for whom they are one of the most prevalent chronic conditions affecting oral health (14). Lower maternal SES, as measured by educational level and income, is associated with a higher incidence of dental caries in children due to its influence on lifestyle, oral hygiene practices, and access to dental care (14, 15). Besides SES, lifestyle factors such as smoking during pregnancy, obesity, and maternal health conditions also influence oral microbiome health among mother and child (14, 16, 17). A study by Falara et al. (18) highlights the harmful effects of perinatal tobacco smoke on the oral microbiomes of both mothers and their infants, increasing the risk of dental caries and tooth decay (18). Similarly, a study of 1,141 mother-child pairs found that maternal obesity and sugar consumption during pregnancy predicted high child sugar intake, which is a major caries risk factor (16). Moreover, high maternal levels of cariogenic bacteria, such as *Streptococcus mutans* and *Lactobacillus* spp., were proven to increase the risk of early childhood caries (14). Together, these findings highlight how the mother’s behaviors and health impact her child’s oral microbiome (14, 15).

To further explore these impacts, this review provides a comprehensive summary of longitudinal changes in the healthy oral microbiome from birth to 5 years of age, focusing on maternal-infant relationships. We highlight studies that have investigated the specialization of the infant oral microbiome, trends in microbial diversity, shifts in dominant taxa, and the evolving similarity between maternal and infant oral microbiomes over time. By synthesizing these findings, we aim to further current knowledge on the processes that influence early oral microbiome development and their clinical implications.

Several narrative reviews have examined similar topics. For example, Azevedo et al. (19) provide useful context on the maternal, environmental, and clinical factors that shape early oral microbiome colonization (19). Their narrative synthesis incorporated studies with diverse designs and age ranges, offering a broad overview of the field (19). Our review differs from this prior work in two key ways. First, while Azevedo et al. (20) included cross-sectional studies ranging to adulthood, we narrowed our scope to longitudinal studies during early childhood (19). This focus allowed direct observation of developmental changes within the same individuals rather than inferring change from cross-sectional age-matched comparisons. This approach is particularly important for our topic considering the nonlinear nature of early oral microbiome development, and it enabled us to draw more precise conclusions regarding the timing of major transitions. Second, while Azevedo et al. (19) included studies published from 2010 to 2020, we concentrated on more recent literature (2018–2025) (19). This allowed our synthesis to reflect advances in sequencing technologies, analytical pipelines, and taxonomic resolution, and to more accurately represent current methodological standards in the field. In these ways, our review builds on the existing foundation of literature, providing a focused and up-to-date synthesis of longitudinal evidence during early childhood.

## LITERATURE REVIEW

### Search strategy

We conducted our search using the following keywords: “mother,” “maternal,” “children,” “infant,” “oral microbiome,” “oral microbiota,” and “longitudinal,” across PubMed, Cochrane Library, and Embase. The keywords used and number of studies returned are shown in Table 1 below.

**TABLE 1:** Search strategies and results from PubMed, Embase, and Cochrane databases

#	Keywords	N	Link	Last search date
1	PubMed: mother, children, oral microbiome, longitudinal	15	https://pubmed.ncbi.nlm.nih.gov/?term=mother+children+oral+microbiome+longitudinal&size=20	6/29/25
2	PubMed: mother, infant, oral microbiome development, longitudinal	12	https://pubmed.ncbi.nlm.nih.gov/?term=mother+infant+oral+microbiome+development+longitudinal&size=20	6/29/25
3	PubMed: maternal, infant, oral bacteria, longitudinal	38	https://pubmed.ncbi.nlm.nih.gov/?term=maternal+infant+oral+bacteria+longitudinal&filter=simsearch2.ffrft&size=20	6/29/25
4	Embase: mother, children, oral microbiome, longitudinal	9	https://www.embase.com/#advancedSearch/resultspage/history.1/page.1/25.items/orderby.date/source.	6/29/25
5	Embase: mother, infant, oral microbiota, longitudinal	16	https://www.embase.com/#advancedSearch/resultspage/history.1/page.1/25.items/orderby.date/source.	6/29/25
6	Cochrane: mother, children, oral microbiome, longitudinal	1	https://www.cochranelibrary.com/search	6/29/25

### Study selection

Studies were initially screened through search filters, which limited the search to studies published in English up to 29 June 2025. Studies were further filtered by manually reviewing abstracts to assess relevance and eligibility. Screening was performed by two reviewers, and no automation tools were used in the process.

**Fig 1:**
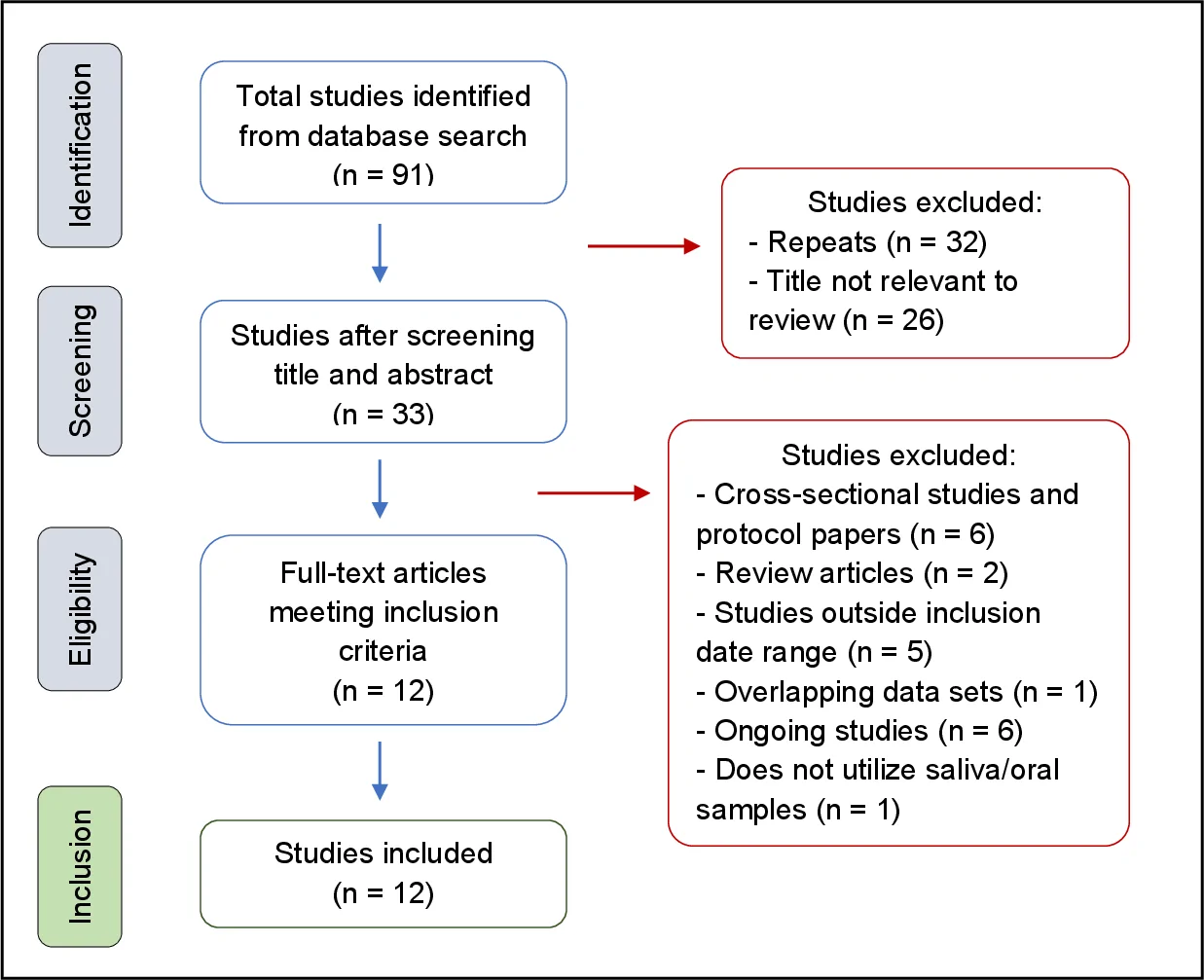
Flow diagram showing search, retrieval, and selection of studies.

Studies were included in the review if they used a longitudinal study design, focused on healthy mother-child dyads, collected saliva or oral microbiome samples, and examined populations aged 0–5 years. Studies published before 2018 were excluded to maintain a manageable scope and to reflect the most recent complete body of literature available. Studies were also excluded if they were review articles, ongoing studies, or included overlapping data sets with previously selected studies. For example, both Kageyama et al. (21) and Kageyama and Takeshita (22) reported on the same cohort, but only the latter was included because it provided more recent conclusions on the data (21, 22). A complete list of excluded studies and reasons for exclusion is included in the supplementary materials. The screening process is summarized by the flowchart (Fig. 1) in the Results section.

### Data extraction

A summary table (Table 2), also shown in the Results section, presents key information from each study, including author, publication date, study design, population demographics, sample types, sequencing methods, taxonomic approaches, and findings. Each study was carefully reviewed, with a focus on any data related to mother-child microbiome similarity, bacterial community composition, changes in microbiome diversity, and associated health outcomes. All relevant findings were recorded on a spreadsheet to organize the connections between studies’ various outcomes. These results were then divided into three groups based on the developmental period they fell into: neonatal (0–4 weeks), infancy (1–12 months), and early childhood (1–5 years). Within each period, results were further categorized by topic, such as microbial diversity, dominant taxa, and maternal-infant microbial similarity. Findings were emphasized if supported by multiple studies, and diverging findings were also discussed.

**TABLE 2:** Summary of included longitudinal studies

Author and year of publication	Title	Study design	Population and sample size	Sample type, sequencing, taxonomy method, and taxonomic resolution	Microbial load vs microbiome analysis (relative abundances/composition data)	Key findings
Sulyanto (10)	The Predominant Oral Microbiota Is Acquired Early in an Organized Pattern	Longitudinal at 12 time points over 12 months	Infants aged 0–12 months and their mothers (*N* = 9 dyads, *n* = 94 samples [69 child, 25 mother]); Healthy children	Saliva; V1–V3 16S rRNA sequencing by Broad Institute; Taxonomy by CORE (resolution up to species level)	Microbiome analysis	Over two-thirds of the infant microbial community was shared with the mother at all time points; Newborns exhibited high variability in oral microbiota, but similarity increased within the first few months; *Streptococcus mitis* was detected in 100% of infants at all ages; Significant increases in richness and diversity were observed after the introduction of solid foods
Ferretti et al. (11)	Mother-to-Infant Microbial Transmission from Different Body Sites Shapes the Developing Infant Gut Microbiome	Longitudinal at five time points: 1 day, 3 days, 7 days, 1 month, and 4 months (stool); two time points: 1 day and 3 days (tongue dorsum)	Infants aged 0–4 months and their mothers aged 26–43 years (*N* = 25 dyads, *n* = 216 samples)	Skin, breast milk, vaginal (mother) and stool, tongue dorsum (both); Shotgun metagenomic sequencing using Illumina HiSeq2500; Taxonomic profiling to species level using MetaPhlAn2 and to strain level using StrainPhlAn	Microbiome analysis	The majority of the newborn oral microbiome was shared with at least one maternal body site; Infants were born with high microbial diversity, which decreased over the first week before stabilizing; At day 1, only 53.6% of bacterial species in the mouth were typical oral microbiome taxa
		Longitudinal at two time points: 4 months and 18 months	Infants aged 4–18 months and their mothers (sample size not provided)	Tongue swab (infants at 4 months, 18 months; mothers at 4 months); 16S rRNA sequencing by PacBio; Taxonomic method not provided (resolution up to species level)	Microbiome analysis	At birth, the oral microbiome was relatively homogeneous with the gut, nasal, and skin microbiota; Similarity to maternal microbiome composition increased between 4 and 18 months; Infants developed one of two distinct profiles by 18 months, with predominance of either *S. salivarius* or *Neisseria*
Williams (23)	Strong Multivariate Relations Exist Among Milk, Oral, and Fecal Microbiomes in Mother-Infant Dyads During the First Six Months Postpartum	Longitudinal at nine time points over 6 months	Infants aged 2 days–6 months and their mothers (*N* = 21 dyads); Healthy dyads	Milk (mother), oral and fecal (infant); V1–V3 16S rRNA sequencing by Integrated DNA Technologies; Taxonomy by RDP database (resolution up to genus level)	Microbiome analysis	*Streptococcus* was predominant in milk and oral samples; Higher similarity was observed between the maternal milk and infant oral microbiomes than the maternal milk and infant fecal microbiomes
Vanzele (20)	Establishment of Oral Microbiome in VLBW Infants During the First Weeks of Life and the Impact of Oral Diet Implementation	Longitudinal at six time points: weeks 1, 2, 3, 4, and before and after oral diet implementation	Preterm infants with VLBW (gestational age 24–25 weeks) and their mothers (*N* = 23 dyads, *n* = 89 samples)	Saliva; 16S rRNA sequencing by Illumina MiSeq; Taxonomy by SILVA (resolution up to genus level)	Microbiome analysis	Microbiome diversity decreased within the first two weeks of life; By week three, the bacterial community was dominated by *Streptococcus, Staphylococcus,* and *Enterobacteriaceae*
Kahharova (24)	Maturation of the Oral Microbiome in Caries-Free Toddlers: A Longitudinal Study	Longitudinal at three time points: 1 year, 2.5 years, and 4 years	Infants aged 1–4 years and their primary caregivers (*N* = 119 dyads, *n* = 925 samples); Healthy, caries-free children	Saliva (both) and dental plaque (infant only); Bacterial V4 16S rRNA amplification and sequencing using Illumina MiSeq; Taxonomic assignment using HOMD (resolution up to species level)	Microbiome analysis + Microbial load (for fungi)	Delivery mode did not impact the composition of the salivary and plaque microbiomes; 60% overlap in salivary microbiome composition between the child and caregiver; The greatest change in the oral microbiome was observed between age 1 year and 2.5 years
Dashper (25)	Temporal Development of the Oral Microbiome and Prediction of Early Childhood Caries	Longitudinal at seven time points: mean ages 1.9 months, 7.7 months, 13.2 months, 19.7 months, 39.0 months, 48.6 months, and 60 months	Children aged 2 months–4 years and their mothers (*N* = 134 dyads, *n* = 762 samples)	Saliva; Bacterial V4 16S rRNA sequencing using Ion Amplicon Library Preparation Fusion Method (Thermo Fisher Scientific); Taxonomic assignment using HOMD (resolution up to species level)	Microbiome analysis	Oral microbiome diversity increased until stabilizing around 48.6 months of age; *Streptococcus mitis* group, *G. haemolysans*, *S. salivarius* group, *H. parainfluenzae*, and *Granulicatella elegans* were the five most abundant OTUs; *S. mutans* was the taxon most strongly associated with early childhood caries (ECC)
Ramadugu (26)	Maternal Oral Health Influences Infant Salivary Microbiome	Longitudinal at four time points: 2 months, 9 months, 12 months, and 24 months	Infants aged 2–24 months and their mothers (>17 years) (*N* = 101 dyads, *n* = 505 samples)	Saliva (mother and infant); V6 16S rRNA sequencing by Illumina HiSeq; Taxonomy by CORE reference database (resolution up to genus level) + targeted qPCR for select species	Relative abundances + Microbial load (qPCR was used for some saliva DNA samples)	Infant oral microbiome diversity and richness increased with age; At two months, C-section infants had significantly higher oral microbiome diversity than vaginally delivered infants; Formula-fed infants had higher diversity than those only fed breast milk
Yama (27)	Oral Microbiota Development in the First 60 Months: A Longitudinal Study	Longitudinal at 13 time points: 1 week, 1, 3, 6, 9, 12, 18, 24, 30, 36, 42, 48, and 60 months	Children aged 1 week–60 months and their parents (*N* = 54)	Saliva and mouth-rinsed water samples; V1–V2 16S rRNA sequencing by Illumina Miseq; Taxonomy by RDP, CORE, NCBI FTP site, and HOMD reference databases (resolution up to species level)	Microbiome analysis	The detection of OTUs consistently detected in >85% of samples increased at approximately 6 months; No significant changes were observed in the weighted UniFrac distance index from 36 to 60 months; By 60 months, the oral microbiota had developed enough to fall within the normal variation seen among adults
Young (28)	Acquisition and Development of the Extremely Preterm Infant Microbiota Across Multiple Anatomical Sites	Longitudinal over the first 60 days of life (time points not specified)	Extremely preterm infants (<26 weeks gestational age) and their mothers (*N* = 7 dyads, *n* = 157 samples); Healthy dyads	Oral and endotracheal secretions, maternal breast milk, stool; 16S rRNA sequencing by Illumina MiSeq; Taxonomy by SILVA (resolution up to genus level)	Microbiome analysis	Microbiota across body sites in extremely preterm infants were most similar immediately after birth and then diverged after 6 weeks
Schulkers Escalante (29)	The Impact of Breastfeeding on the Preterm Infant’s Microbiome and Metabolome: A Pilot Study	Longitudinal at four time points: two during tube feeding, two during breastfeeding	Infants aged up to 4–5months and their mothers (*N* = 11 dyads, *n* = 126 samples); Healthy dyads	Stool, saliva, and breast milk; 16S rRNA sequencing by Illumina NovaSeq6000; Taxonomy by Web of Life (resolution reported up to genus level)	Microbiome analysis	Delivery mode did not significantly influence the salivary microbiome for preterm infants; After the initiation of breastfeeding, *Streptococcus* increased and *Staphylococcus* decreased
Takahashi (30)	Periodontal Pathogen Colonization in Young Children by PCR Quantification – A Longitudinal Survey	Longitudinal at four time points: 6 months, 12 months, 18 months, and 24 months	Infants aged 0–2 years and their mothers (*N* = 37 dyads; sample count not provided)	Saliva and oral biofilm (both); Species-specific PCR amplification using Perkin Elmer GeneAmp PCR System 2400 and gel electrophoresis; Taxonomic database not provided (resolution up to species level)	Microbial load	Positive correlation was detected in the occurrence of periodontal pathogens between mothers and children; Hygiene, dietary habits, and parental attitudes toward dental hygiene appeared to influence children’s oral health

This review was written in accordance with the Preferred Reporting Items for Systematic Reviews and Meta-Analyses (PRISMA) 2020 checklist (31). We included a compliance checklist in supplemental materials for the systematic review in the supplement material to indicate where each item was presented in the manuscript. After drafting, minor edits were made using AI-assisted language tools to improve grammar, clarity, and flow. All edits were reviewed and approved by the authors. No AI tools were used for study selection, data extraction, analysis, or interpretation of findings.

## SUMMARY OF STUDIES BY AGE

The initial search returned 91 studies, with 12 meeting the criteria for inclusion in the analysis. The screening process is summarized in Fig. 1.

**Fig 2:**
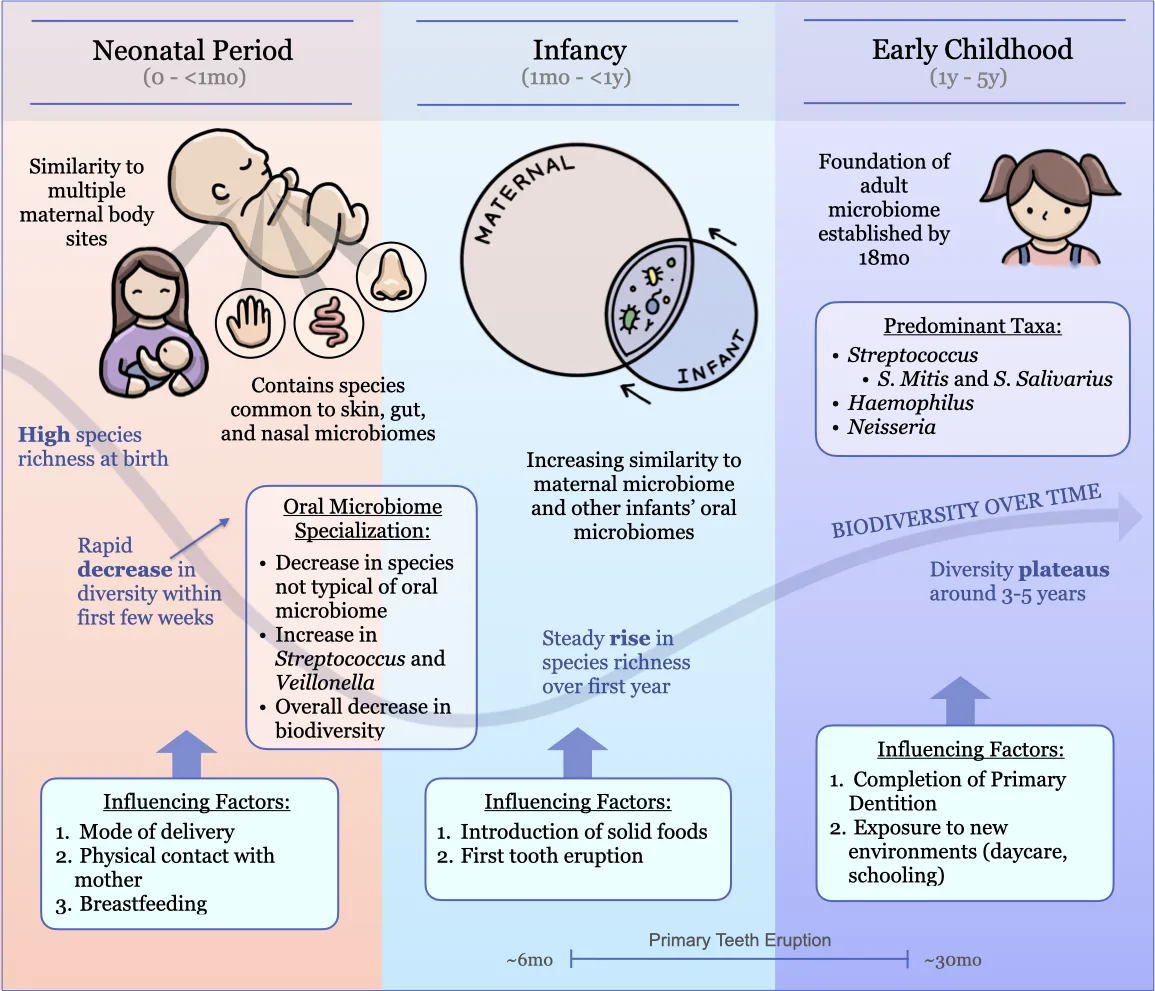
Oral microbiome development from birth to 5 years old.

To simplify the presentation of results, we organized findings into three developmental stages: the neonatal period (0–4 weeks), infancy (1 month–1 year), and early childhood (1–5 years). Shortly after birth, the oral microbiome is highly dynamic and susceptible to rapid changes (11, 20, 22, 23). Thus, the first section of results focuses on the neonatal period to capture any short-term changes that occur in the beginning of oral microbiome development. Following this period, microbial trends become more gradual, so the next section expands to encompass the entire first year after one month. We chose the first year as a benchmark because it represents the first recommended dental visit for children and is when the infant oral microbiome begins to resemble the foundations of an adult microbiome. In the rest of the Results section, we present the general trends until 5 years of age, as the microbiome begins to stabilize and changes are less distinct (10, 22–26). We chose to end our analysis at 5 years, as this is when the oral microbiome begins to fall within the normal variation seen among adults (27). This organizational structure is outlined by the timeline shown in Fig. 2.

### Neonatal period (0–4 weeks)

The first 4 weeks after birth represent a critical period for the establishment of the oral microbiome. During this time, the early colonization of the infant oral microbiome is heavily influenced by the maternal microbiome, since the mother provides one of the first points of exposure to the outside world. Current research indicates that, immediately after birth, the infant oral microbiome exhibits a significant overlap with several maternal body sites and that specialization of the oral microbiome begins within the first week (10, 11).

In a longitudinal study of 25 mother-infant pairs, Ferretti et al. (11), using shotgun metagenomic sequencing of tongue dorsum samples, found that a substantial portion of the infant oral microbiome is maternally derived (11). They noted that 77.6% of species present in the infant oral microbiome at day 1 postpartum were also found in at least one maternal body site (oral, skin, vaginal, breast milk, or stool), with this proportion increasing to 95.4% by day 3 (11). Despite this overlap, the infant oral microbiome remained less complex than its maternal counterpart (11). Although the shared species accounted for high proportions of the infant oral microbiome, they represented only 5.7% and 6.6% of the total abundance in the maternal tongue dorsum at days 1 and 3, respectively (11). These findings show that while maternal seeding plays a crucial initial role in infant oral microbiome composition, the infant’s oral microbiome is still rudimentary compared to the maternal oral microbiome. The findings of Sulyanto et al. (10) further support Ferretti et al. (11) (10, 11). Using a longitudinal 16S rRNA sequencing analysis of saliva samples from nine mother-infant pairs over the first year of life, they found that over 85% of the total microbial abundance in infants was shared with their mothers (10). These data reinforce the conclusion that the mother is a primary source of early colonizers in the infant oral microbiome.

Another noteworthy finding was that the neonatal oral microbiome is unspecialized, demonstrating high species richness and similar composition to the microbiota of other body sites. Ferretti et al. (11) noted that, at day 1, 46.4% of bacteria identified in infant tongue dorsum samples belonged to species not typically detected in adult oral microbiota (11). This was supported by Sulyanto et al. (10), who identified many microbial species commonly associated with the gut, skin, and external environments in newborns’ mouths (10). Additionally, at birth, Kageyama and Takeshita (22) reported a high abundance of *Propionibacterium* and *Lactobacillus*, which are not typical of adult oral microbiota, and further determined that the oral microbiota is relatively homogeneous with the gut, nasal, and skin microbiota (22). Young et al. (28) observed similar patterns in seven extremely preterm infants and their mothers, reporting that microbial diversity was similar across oral, stool, and breastmilk samples at 0–6 weeks (28). Thus, there is ample evidence indicating the oral microbiome is unspecialized at birth for both term and preterm infants. This initial lack of specialization is the starting point from which the oral microbiome develops site-specific compositions and establishes the foundation for a mature microbial community.

Following the initial unspecialized state at birth, the infant oral microbiome undergoes rapid specialization within the early days and weeks of life, as shown by the timeline in Fig. 2. Ferretti et al. (11) reported that infant oral microbiome diversity decreased over the first week postpartum and recovered over time (11). This decrease was characterized by a dramatic reduction in the proportion of bacterial species that were not typical of adult oral microbiota, which fell from 46.4% at day 1 to 3.1% at day 3 (11). Similar results were observed in 16S-based studies of saliva samples from very low birth weight (VLBW) infants and extremely preterm infants (20, 28). Vanzele et al. (20), who studied 23 infants with very low birth weight, observed a decrease in microbiome diversity within the first 2 weeks in 23 VLBW infants (20). In addition, Young et al.’s (28) study of preterm infants reported increasing differences in diversity between breast milk, oral, and stool microbiota around 6 to 8 weeks, which indicates independent microbiome development in different body sites (28).

Kageyama and Takeshita (22) provided additional insight into the specific taxonomic shifts that occur during this rapid specialization (22). Their analysis of infant tongue swab samples revealed a proliferation of *Streptococcus* and *Veillonella* within the first 6 weeks of life (22). Since these species are characteristic of mature oral microbiomes, their increase reflects the establishment of a foundational oral microbiome. Indeed, Sulyanto et al. (10) determined that oral bacterial community compositions among children became increasingly similar to one another within the first few months of life, indicating a progression toward a more uniform and typical oral microbial community (10).

Another notable observation about the neonatal oral microbiome was the critical role it played in influencing the gut microbiome during the first few months of infancy. Using SourceTracker 2 software to model the contribution of oral bacterial communities to fecal bacterial communities in 21 infants, Williams et al. (23) estimated that approximately 21% of the infant fecal microbiota originated from the oral cavity at day 2 postpartum, which then increased to 66% by 5 months (23). This indicates that the oral cavity may be a significant source of microbial seeding for the infant gut microbiome. Ferretti et al. (11) further noted that the extent of oral microbiome seeding into the gut is more significant in infants compared to adults (11). On day 1, species shared between tongue dorsum and stool samples accounted for 24.85% of the total microbial abundance in infant stool samples, but only 0.96% in maternal stool samples (11). This difference may reflect the simpler microbial communities in infants, which could facilitate the presence of oral taxa in the gut. Moreover, there may be a higher impact on the child’s oral and gut microbiomes during the early stages of life because the child’s immune system is not yet fully matured (32). On the other hand, the more complex and established microbiota of adults may inhibit the survival of oral microbes in the gut. Oral-gut microbiome connections may help explain the oral microbiome’s importance in systemic immune health (4).

Several studies also investigated the impact of delivery methods on the early development of the oral microbiome although results remain inconclusive. In one study, Kahharova et al. (24) investigated the maturation of the oral microbiome in 119 caries-free children, including 77 delivered vaginally and 42 delivered by C-section (24). They found no significant differences in salivary microbiome composition between the two groups, suggesting that delivery mode did not play a significant role in oral microbiome development for that population (24). Similarly, Schulkers Escalante et al. (29) reported that delivery methods did not significantly influence the salivary microbiome of preterm infants (29).

On the other hand, Ramadugu et al. (26) reported differences in microbial diversity and composition between vaginally delivered and C-section infants (26). They analyzed salivary microbiomes in 101 infants, including 69 delivered vaginally and 30 via C-section, and found that the salivary microbiomes of C-section infants were significantly more diverse at 2 months of age than those delivered vaginally (26). The study also noted taxonomic differences, with higher relative abundances of *Streptococcus, Rothia,* and *Gemella* in C-section infants, and higher abundances of *Granulicatella, Porphyromonas,* and *Haemophilus* in vaginally delivered infants, after adjusting for age and geographic differences (26). These differences were no longer detected by 12 months, implying that the effect of delivery mode on oral microbiota may not be permanent (26).

Given the diverging findings, the evidence remains inconclusive on whether and how delivery method influences the early oral microbiome. Further research is needed to determine the contribution of delivery mode to early oral microbiome development.

### Infancy (1–12 months)

The development of the oral microbiome continues throughout the first year of life, characterized by an increase in species richness and establishment of a common community composition. These changes bring the infant community composition closer to the oral microbial profiles of their mothers and other children.

After the initial decrease in species richness that was observed in the first month, richness increases again, indicating the beginnings of colonization and diversification (as shown in Fig. 2). Sulyanto et al. (10) reported a mean species richness of 30 between 0 and 2 months of age, which increased to 33 by 10–12 months (10). Similarly, Williams et al. (23) reported a species richness of 23 at day 2, which increased to 29 by 6 months (23). Therefore, the first year of life is characterized by an increase in oral microbial richness. However, the infant microbiome does not yet reach the same level of complexity as its adult counterpart. Sulyanto et al. (10) noted that species richness at 10–12 months was still substantially lower than that of maternal microbiomes, which exhibited a mean richness of 83 species compared to only 33 in infants (10).

As the oral microbial community diversifies, its composition increasingly resembles typical adult microbiomes and converges across infants. Both Kageyama and Takeshita (22) and Williams et al. (23) observed increasing similarity between infant and maternal microbiomes between 4 and 18 months (22, 23). Furthermore, multidimensional scaling analyses by Sulyanto et al. (10) and Williams et al. (23) both demonstrated that infant oral microbiomes became increasingly similar to those of other infants over the first year of life (10, 23). In addition to demonstrating convergence, studies provided insights into the relative abundances of taxa composing the microbial community profile. A consistent finding was that *Streptococcus* represents the most abundant genus among infant oral samples (10, 20, 23). In fact, Sulyanto et al. (10) detected members of the *Streptococcus mitis* group in 100% of infants studied across all time points from birth to 12 months (10). *Staphylococcus* was also frequently identified as an abundant taxon though the next few most predominant taxa varied between studies (10, 20, 23). For example, Vanzele et al. (20) identified Enterobacteriaceae as the next most abundant family in preterm infant oral samples, whereas Williams et al. (23) found the genera *Gemella, Rothia,* and *Veillonella* to be the next most abundant (20, 23). Breast milk also exhibited these same five most abundant taxa, exhibiting greater similarity with oral samples than fecal samples did (23).

Maternal-infant connections continued to play a key role in infant oral microbiome health throughout the first year. This trend is supported by Takahashi et al. (30), who reported a strong positive correlation between the occurrence of periodontal pathogens in mothers and their children at 6 and 12 months (30). Therefore, infant oral microbiomes appear to be shaped by maternal oral health.

Another notable factor influencing oral microbiome development in the first year of life was feeding practices. Various feeding practices affected microbial colonization, including breastfeeding vs formula feeding, the transition from tube to oral feeding for VLBW infants, and the introduction of solid foods (10, 20, 26, 29). Ramadugu et al. (26) compared the oral microbiomes of 18 exclusively breastfed infants with 24 exclusively formula-fed infants and determined that the breastfed group exhibited lower Shannon diversity (2.77 vs 2.92 Shannon diversity at 2 months, respectively) (26). However, by 12 months of age, this difference in diversity diminished, suggesting that a formula diet may influence salivary microbiome diversity in early infancy but does not have significant consequences beyond the first year (26).

Two studies focused on the effects of the transition from tube to oral feeding in preterm infants. Schulkers Escalante et al. (29) found increases in the relative abundances of *Streptococcus* and *Klebsiella* after the transition to oral feeds in a cohort of 11 preterm infants (29). In a similar study of 23 preterm infants, Vanzele et al. (20) observed that relative abundances of *Streptococcus* and *Staphylococcus* remained high after the introduction of an oral diet, while the relative abundance of Enterobacteriaceae decreased (20). Together, these findings indicate that the transition to oral feeding in preterm infants is associated with notable shifts in oral microbial composition.

The introduction of solid foods has also been shown to cause changes in the oral microbiome. Sulyanto et al. (10) investigated nine infants that transitioned from milk to solid foods at around 7 months of age and reported significant increases in Shannon diversity and rarefied species richness (10). These results further demonstrate the contribution of changes in diet to the diversification of the oral microbial community.

### Early childhood (1–5 years)

During the next 4 years of life, the oral microbiome continues to rapidly develop and diversify before ultimately stabilizing into the foundation of the adult oral microbiome. Although the child’s oral microbiome increasingly resembles the mother’s, it remains comparatively less diverse throughout early childhood.

A study by Dashper et al. (25) investigated oral microbiome development in 134 children at seven time points between 2 months and 4 years of age (25). The study examined the “core oral microbiome,” which they defined as the set of taxa that were detected in 90% or more infants at any given time (25). In infants, the core oral microbiome developed from seven operational taxonomic units (OTUs) at 2 months to 32 OTUs at 20 months (25). Despite the increase in diversity, maternal core oral microbiomes were still comparatively more complex, averaging 54 taxa (25). In terms of overall bacterial diversity, the total number of OTUs in infant oral microbiomes increased from an average of 31 at 2 months to an average of 84 at 39 months (25). Similar trends were observed by Ramadugu et al. (26), who documented increases in Shannon diversity and richness from 2 months to 2 years although specific measurements at different time points were not reported (26). Kahharova et al. (24) reinforced this pattern, recording a substantial increase in oral microbiome diversity between 1 and 2.5 years, with the number of total OTUs increasing from 235 to 369 per sample (24). They reported that similarity between child and caregiver oral microbiomes increased significantly from 1 to 2.5 years, with a 60% overall overlap in salivary microbial community composition between mother and child (24). At all time points, child oral microbiome diversity was lower than maternal diversity, which was also consistent with Dashper et al.’s (25) findings (24, 25). These studies highlight the first 3 years of life as a critical phase for oral microbiome diversification.

Despite changes in overall diversity, the dominant genera in the child oral microbiome stayed consistent. Kageyama and Takeshita (22) identified a profile predominated by *Streptococcus salivarius* and *Neisseria* at 18 months of age, and Kahharova et al. (24) found *Streptococcus, Haemophilus*, and *Neisseria* to be most abundant between ages 1 and 4 (22, 24). Dashper et al. (25) further emphasized the predominance of *Streptococcus mitis*, describing it as the most abundant OTU in saliva “by far” although its relative abundance declined by age 4 (25). Notably, at around 49 months, the *Streptococcus mitis* group was twice as abundant in children’s saliva compared to maternal saliva (25). Other compositional shifts included increases in *Fusobacterium, Actinomyces*, and *Corynebacterium*, as reported by Kahharova et al. (24).

The compositional shifts occurring throughout years 1–3 are then followed by a plateau phase, in which much fewer changes are observed in oral microbiome community composition. Despite differences in study design, sample type, and sequencing approach, three studies— Dashper et al. (25), Kahharova et al. (24), and Yama et al. (27)—each reported no significant change in oral microbiome diversity from approximately 3 to 5 years of age (24, 25, 27). By 5 years, according to Yama et al. (27), the oral microbiome had matured enough to fall within the normal variation seen among adults (27). This demonstrates the closing of the development period for the oral microbiome and the establishment of a foundation for the full adult microbiome.

Two studies focused on tooth eruption in early childhood to investigate whether the emergence of enamel surfaces would impact the oral microbiome by introducing new habitats for bacterial colonization. Although Sulyanto et al. (10) posited that there was no significant change in the oral microbiome after the first tooth eruption, Kahharova et al. (24) reported significant differences in alpha and beta diversities between the microbial profiles of predentate children compared to the saliva of children with five or more teeth (10, 24). Taken together, these findings suggest that while the first tooth eruption may not noticeably impact the oral microbiome, cumulative eruption (≥5 teeth) can be associated with detectable differences (10, 24). Further research is needed to confirm the role tooth eruption plays in shaping the oral microbiome.

Dashper et al. (25) also explored whether maternal oral microbiomes could predict the risk of early childhood caries (ECC) (25). They identified *S. mutans* as the taxon most strongly associated with ECC, along with *S. sobrinus* and *Veillonella parvula* (25). Meanwhile, *Prevotella nigrescens*, *Leptotrichia* spp., and *Actinobaculum* 12B759 were less abundant in children with oral disease (25). However, maternal *S. mutans* levels did not differ significantly between mothers of children with or without ECC, demonstrating that the abundance of *S. mutans* in mother’s saliva is not confirmed to be a reliable predictor of ECC risk in their children (25).

## FUTURE SUGGESTIONS

### Overview of early oral microbiome development

The findings reviewed in this paper provide valuable insights into trends in diversity, relationships with maternal microbiomes, and changes in community composition of the oral microbiome during the first 5 years of life. Among the 12 studies, we identified several common themes, which together provide the general framework for early oral microbiome development. Starting from birth, maternally derived bacterial strains make up the majority of the newborn’s oral microbiome (10, 11). At this point, infants have highly diverse and unspecialized oral microbiomes that resemble microbiota from the skin, gut, and nasal cavity (10, 11, 22, 28). The oral microbiome then undergoes rapid selection in the first few weeks of life, as the relative abundances of bacterial species typically found in adult oral microbiomes increase (11, 20, 22, 23). After the initial phase of selection, diversity and similarity to maternal oral microbiomes increase steadily, before stabilizing around 3 years of age (10, 22–26). By this time, the child’s oral microbiome represents a less complex version of the full adult microbiome (24, 25). Dietary practices during the first year of life, such as methods of feeding (breastfeeding or formula) and the introduction of solid foods, have been shown to impact oral microbiome diversification (10, 20, 26, 29).

### Factors contributing to differences across studies

Despite a number of shared findings, the literature also contained some inconsistencies. To better contextualize these varied findings, we propose several hypotheses to explain how differences in methodology and study design may have contributed to diverging results.

First, studies disagreed on whether the mode of delivery has a significant impact on oral microbiome development. Ramadugu et al. (26) observed a significant difference in the oral microbiomes of vaginally delivered infants and C-section infants, while Schulkers Escalante et al. (29) and Kahharova et al. (24) detected none (24, 26, 29). Study design differences could explain this disagreement; though both studies used 16S rRNA sequencing, they differed in sample collection timing. Kahharova et al. (24) began collecting samples at one year of age, which may have caused a blind spot in the earlier months (24). On the other hand, Ramadugu et al. (26) began sampling at 2 months and found that differences in diversity and composition mostly disappeared by 12 months (26). From this, we hypothesize that any influence of delivery mode on the oral microbiome may be temporary and most evident in only the earliest months of life. To obtain clearer evidence, future studies should focus on mode of delivery using longitudinal sampling that begins immediately after birth and continues through early childhood. Such continuous analysis would help identify and characterize any associated impacts with greater certainty.

Another area in which results varied was the predominant taxa identified during infancy. Although three studies agreed that *Streptococcus* and *Staphylococcus* were the most abundant genera in the infant oral microbiome, Vanzele et al. (20) and Williams et al. (23) differed in their reports of the next most abundant taxa, with the former reporting Enterobacteriaceae and the latter reporting *Gemella, Rothia*, and *Veillonella* (10, 20, 23). We propose two possible explanations for this divergence, the first of which is differences in study populations. Vanzele et al. (20) examined extremely preterm VLBW infants, who commonly undergo frequent antibiotic exposure, delayed oral feeding, and increased contact with hospital-associated microbes (20, 33). These conditions may explain the higher relative abundance of Enterobacteriaceae, which are opportunistic colonizers and often have antibiotic resistance mechanisms (33). In contrast, Williams et al. (23), studied healthy mother-infant dyads and spanned a longer time period (23). In this population, it would be expected to observe higher abundances of common oral genera such as *Gemella, Rothia*, and *Veillonella*, given the absence of frequent antibiotic exposure, delayed oral feeding, and prolonged hospitalization (23, 33). Second, methodological differences in taxonomic classification may further contribute to these differing results. Vanzele et al. (20) used the SILVA database, while Williams et al. (23) used the RDP database (20, 23). SILVA and RDP differ significantly in size, resolution, and taxonomic content (34). Therefore, database choices may have influenced the taxa identified by these two studies. Together, these biological and methodological differences may explain the divergent taxa reported across studies.

Lastly, we examine discrepancies between the predominant taxa reported by Kageyama and Takeshita (22) and Dashper et al. (25) in early childhood (22, 25). While Kageyama and Takeshita (22) identified *Streptococcus salivarius* as a dominant taxon, Dashper et al. (25) reported that *Streptococcus mitis* was predominant “by far” (22, 25). One possible explanation for this conflict could be sampling sites used, as Dashper et al. analyzed saliva samples, whereas Kageyama and Takeshita sampled the tongue dorsum (22, 25). Given these methodological differences, the reported taxa may reflect site-specific microbial communities rather than overall oral microbiome composition. In this context, *S. mitis* is likely better adapted to saliva-associated communities, where it circulates more loosely, whereas *S. salivarius* may be more stably associated with a single oral habitat, such as the tongue surface. This interpretation is consistent with findings from Sulyanto et al., who also analyzed saliva samples and identified *S. mitis* as a predominant taxon, detecting it in 100% of samples and at higher prevalence than *S. salivarius* (10). Future studies that sample multiple oral sites within the same individuals would help clarify habitat preferences and ecological roles of common oral taxa.

Overall, methodological variance likely contributes to many of the inconsistencies observed across studies.

### Methodological limitations of the review

This review has several methodological limitations that may have affected the evidence included. Although study screening was conducted carefully, studies were selected by two reviewers, which may have caused selection bias and reduced objectivity. Furthermore, there may have been relevant data from non-English sources that were not captured due to the language restriction applied during the search. Another possible limitation was the choice of keywords for the search, which may not have encompassed any studies that used variant terminology and, therefore, fell out of scope.

### Gaps in the literature and future research directions

Overall, the existing literature provides a useful foundation, but there is still a need for larger and more focused studies on the topic. There were relatively few studies returned by our search, which demonstrates how limited the scope of research is on maternal-infant interactions in early oral microbiome development. Of the 12 studies we examined, only 4 had a sample size of more than 50 mother-child pairs, and all these studies except Yama et al. (27) were published over 4 years ago (27). Furthermore, much of the current research is primarily observational. Most of the results describe trends in diversity and community profile composition without exploring the potential lifestyle and behavioral factors that might influence them. For example, it is still largely unclear how factors like dietary habits, exposure to maternal illness, and household environment influence the oral microbiome. To address these knowledge gaps, we suggest that future studies prioritize using larger sample sizes and incorporate metadata about external and environmental influences in their analysis. This could be done through questionnaires or interviews collecting data about participants’ lifestyle habits and environmental exposures. These recommendations would allow researchers to go beyond preliminary observation and begin to identify the driving factors that impact the oral microbiome in early childhood.

Antibiotic exposure during pregnancy and breastfeeding is a crucial factor that affects mother–infant microbial interactions and has not been adequately studied in the reviewed literature. This may contribute to inconsistent findings regarding the effects of delivery mode (24, 26, 29). The negative impact of antibiotic use to treat bacterial infections during pregnancy may have long-term consequences, including the spread of antibiotic resistance and alterations in the function of the gut microbiota (35). However, its impact on the oral microbiome and oral health is not clear (35). Cross-sectional studies have indicated differences in both taxa and metabolic function of the initial neonatal oral microbiome at birth associated with antibiotic treatment during pregnancy (36–38). Longitudinal studies are needed to investigate the long-term, complex impact of antibiotic use on the onset and development of the neonatal oral microbiome during early childhood, as well as the timing of exposure during pregnancy and breastfeeding. There are varying clinical guidelines regarding antibiotic use during pregnancy. According to the most recent national consensus statement (January 2026) (39), several antibiotics are considered appropriate for use during pregnancy when clinically indicated, including amoxicillin, penicillins, cephalosporins, clindamycin, and metronidazole. In contrast, ciprofloxacin, clarithromycin, levofloxacin, and moxifloxacin are recommended to be avoided during pregnancy due to potential fetal risks. Tetracyclines are contraindicated and should never be used during pregnancy.

Expanding knowledge on these factors is the crucial next step toward developing microbiome-targeted therapeutic interventions and translating this research into clinical application. Research on microbiome-targeted interventions for the gut has already begun to explore the potential for prebiotic and probiotic supplements for gut microbiome health in pregnant women and neonates (40, 41). One such study was able to successfully modulate the gut microbiome in preterm neonates, seeing a significant difference in the gut microbiota after administering probiotics (41). Given more research on diet and the oral microbiome, scientists can begin to test the feasibility for similar interventions for oral microbiome health.

Continuing research in this area is important because emerging new technologies enable increased precision and larger, more comprehensive studies. For example, PacBio long-read sequencing, made possible by third-generation sequencers, offers advantages over the short-read Illumina sequencing that is used in most current longitudinal oral microbiome studies (42). Long-read sequencing remains uncommon in oral microbiome research; among the studies we reviewed, only one utilized PacBio, while seven used Illumina (Table 2). Most studies used short-read 16S rRNA sequencing targeting certain regions, and the resolution may reach only the genus level. The limitation of low-resolution 16S-based microbiome analysis is that it may not be able to detect strain-level differences (43). In addition, 16S rRNA sequencing can analyze only bacteria, which are just one part of the oral microorganism community (approximately 1,000 species of bacteria, fungi, viruses, archaea, and protozoa) (44). With the development of technology, more research is needed to study the full scope of oral microorganisms in the mouth and their influence on oral microbiome development and transmission between mother and infant in early childhood.

In addition, advances in artificial intelligence (AI) offer powerful tools for analyzing large data sets, such as microbial community profiles, and could help monitor shifts in the microbiome and host-microbe interactions (45). For example, machine learning, an implementation of AI, has been used to study tooth decay by analyzing patterns in oral bacteria data (3). While AI is not yet widely used in dental care, machine learning combined with advanced computational analysis has the potential to improve the accuracy and efficiency of oral microbiome studies. Furthermore, an advanced cellular imaging technique called fluorescence *in situ* hybridization (FISH) allows for more detailed visualization of how human and bacterial cells are spatially organized within the mouth (46). This method may help researchers investigate how bacterial communities interact and shape microbial development in early life. Together, these emerging technologies have promising applications for future research aimed at deepening our understanding of early oral microbiome development.

## CONCLUSION

This review highlights important trends in the early development of the oral microbiome and the pivotal role of maternal influences. However, substantial gaps remain, and further research is needed to clarify the factors that shape oral microbiome development and to determine their long-term health implications.

This review synthesizes current evidence on early oral microbiome development and demonstrates that the first years of life represent a critical window of microbial establishment shaped by maternal transmission, early environmental exposures, and infant feeding practices. Across studies, a consistent developmental pattern emerges: newborns harbor highly diverse and nonspecialized oral microbial communities that rapidly undergo selection toward oral-adapted taxa, gradually increasing in similarity to maternal and adult-like microbiomes and stabilizing by approximately 3 years of age. These findings underscore the foundational role of maternal influence and early-life exposures in structuring the oral ecosystem during infancy and early childhood.

At the same time, this review highlights substantial heterogeneity across studies, much of which appears attributable to differences in study design, population characteristics, sampling strategies, sequencing technologies, and taxonomic databases. Variability in reported associations with delivery mode, predominant taxa, and early microbial composition underscores the need for standardized methodologies and longitudinal study designs that begin at birth and incorporate multiple oral sampling sites. Significant gaps remain regarding the influence of antibiotic exposure, diet, household environment, and broader social and behavioral factors on oral microbiome development.

Addressing these gaps through larger, more comprehensive, and technologically advanced studies, including long-read sequencing, multi-kingdom analyses, and AI-driven analytical approaches, will be essential for advancing mechanistic understanding and translating early oral microbiome research into targeted preventive and therapeutic strategies that support lifelong oral and systemic health.

## SUPPLEMENTAL MATERIAL

Supplemental material iai.00585-25-s0001.docx PRISMA 2020.

Table S1 iai.00585-25-s0002.pdf Excluded studies.

ASM does not own the copyrights to Supplemental Material that may be linked to, or accessed through, an article. The authors have granted ASM a non-exclusive, world-wide license to publish the Supplemental Material files. Please contact the corresponding author directly for reuse.
