# Supplementary material for: Maternal influences on oral microbiome development and implications for early childhood health: a systematic review
Source: Infect Immun. 2026 Mar 3;94(4):e00585-25. doi: 10.1128/iai.00585-25 (PMC13047901; doi:10.1128/iai.00585-25)
Supplement: Table S1 — Excluded studies. [file iai.00585-25-s0002.pdf]

Search # (as listed in Table 0)	Author and Year	Reason(s) for Exclusion *Repeated studies not shown (n = 32)
1. PubMed	Azevedo 2023	Review article
	Xiao 2018	Title not relevant- focuses on subjects with severe ECC
	Takahashi 2017	Overlapping data set with Takahashi 2018
	Seedat 2017	Review article
	Sprockett 2020	Title not relevant- highly specific population
	Susic 2020	Ongoing study (results not yet available)
	Moosavi 2019	Cross-sectional study
	Chortatos 2025	Title not relevant- focuses on behavioral temperament
2. PubMed	Seifert 2025	Cross-sectional study
	Biagi 2018	Title not relevant- focuses on breastmilk
	Ward 2018	Title not relevant- mycobiome focus
	Klein 2004	Study outside inclusion date range
3. PubMed	Dominguez-Bello 2016	Ongoing study (results not yet available)
	Davis 2024	Title not relevant- focuses on gut microbiome
	Koskela 2025	Title not relevant- not related to microbiome
	Mandolo 2023	Title not relevant- breastmilk microbiome focus; ongoing study
	Hashemipour 2023	Ongoing study (results not yet available)
	Chu 2017	Study outside inclusion date range
	Flaviani 2021	Title not relevant- cervicovaginal microbiota
	Amir 2011	Title not relevant- focuses on breast pain; study outside inclusion date range
	Xu 2025	Title not relevant- focuses on milk microbiome
	Deshpande 2024	Title not relevant- focuses on skin microbiome
	Rinne 2025	Title not relevant- focuses on Epstein–Barr virus
	Oswald 2008	Title not relevant- not related to oral microbiome development; study outside inclusion d
	Church 2019	Title not relevant- not related to microbiome
	Brummaier 2019	Title not relevant- focuses on pregnant mothers only
	Stowell 2014	Title not relevant- Cross-sectional; study outside inclusion date range
	Koskimaa 2017	Title not relevant- focuses on human papillomavirus in placenta; study outside inclusion
	Sarkola 2008	Title not relevant- focuses on human papillomavirus in placenta; study outside inclusion
	Farquhar 2011	Title not relevant- focuses on HIV-1 virus; study outside inclusion date range
	Corwin 2017	Study outside inclusion date range
	Mbuya 2015	Title not relevant- focuses on fecal microbes; study outside inclusion date range
	Warren 2008	Study outside inclusion date range
	Ye 2020	Title not relevant- antibody focus
	Momeni 2018	Cross-sectional study
	Warren 2009	Study outside inclusion date range
4. Embase	Osagie 2025	Ongoing study (results not yet available)
	Pena 2020	Cross-sectional study
	Korpela 2019	Does not use saliva/oral samples
	Susic 2019	Protocol paper
5. Embase	Christensen 2023	Title not relevant- stool microbiome
	Feiko 2023	Title not relevant- does not mention oral microbiome
	Dempsey 2023	Ongoing study (results not yet available)
	Roager 2022	Ongoing study (results not yet available)
	Kaan 2020	Cross-sectional study
	Moya-Alvarez 2018	Title not relevant- gut microbiome focus
	Williams 2017	Title not relevant- does not focus on maternal influence
6. Cochrane Library	N/A	Repeated study
